# Impacts of Waste Rubber Products on the Structure and Properties of Modified Asphalt Binder: Part II—Rubber Reclaim

**DOI:** 10.3390/ma17205091

**Published:** 2024-10-18

**Authors:** Svetlana Obukhova, Angelina Budkina, Evgeny Korolev, Vitaly Gladkikh

**Affiliations:** 1 Department of Urban Planning, Institute of Architecture and Urban Planning, National Research Moscow State University of Civil Engineering, Moscow 129337, Russia; 2 Scientific and Educational Center “Nanomaterials and Nanotechnologies”, National Research Moscow State University of Civil Engineering, Moscow 129337, Russia; 3 Research Center «MGSU Stroy-Test», National Research Moscow State University of Civil Engineering, Moscow 129337, Russia

**Keywords:** rubber reclaim, modified asphalt binder, structure, compatibility, stability, solubility, performance grade

## Abstract

The issue of forming a reliable and stable structure of a crumb-rubber-modified binder is an important scientific and technical task. The authors supplemented existing concepts of the mechanism of effective interaction with rubber crumb by introducing a preliminary first stage: controlled partial physical destruction of rubber crumb—producing rubber reclaim. Proposed physical methods of rubber crumb destruction include high shear force (roll mills), high temperature, and a plasticizing medium. The controllability and degree of devulcanization of rubber were determined by acetone-chloroform extraction in different time intervals. The degree of devulcanization of rubber in the rubber reclaim was found to be 22 ± 0.24%, with stability over 14 days. It was found that the size of the particles of the rubber reclaim in the bitumen is less than 2 µm. The properties of the structure of the binder modified with rubber reclaim, characterizing the stability and sustainability, have been studied and established. The developed modified binders are stable in storage. Rheological parameters of the structure characterizing intermolecular interaction, such as shear stability for original and RTFOT-aged, modified bitumen, meet requirements of the state standard at test temperature 64 °C. The elastic structural component of the crumb-rubber-modified binder, as indicated by the relative irreversible deformation parameter *J*_3,2_, does not exceed 2.6 kPa (<4.5 kPa) at 64 °C. Additionally, it was determined that the rheological structural parameter for fatigue resistance, which characterizes the durability of road pavement under intensive operational conditions, does not exceed 4699 kPa (<5000 kPa) at 16 °C. The use of 10% rubber reclaim combined with waste frying oil provided the opportunity to obtain a modified binder with a stable and sustainable structure without the introduction of additional stabilizers and agents. Test results showed that the overall performance characteristics of the modified binder meet the 64(S)-40 grade standards.

## 1. Introduction

**Figure 1:**
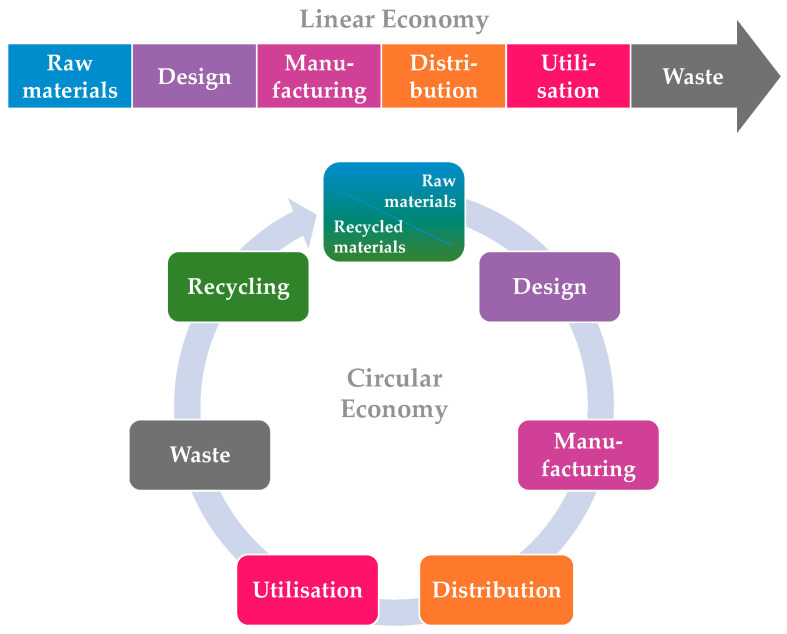
Comparison of linear and circular types of economy.

One of the most important goals in the design and construction of roads is to ensure the best technical and economic performance without compromising quality. Therefore, an important direction for the sustainable development of any country is the search for and development of materials and technologies that provide optimal technical and economic indicators, thereby enhancing the reliability and durability of roads. Sustainable development is a global concept aimed at preserving the planet’s resources for future generations. The Sustainable Development Goals adopted by the UN in 2015 provide a strategy to address global environmental, economic, and social challenges [1]. To adhere to this principle, it is necessary to transition from a linear economy to a circular economy, as shown in Figure 1. This involves shifting from the “take, make, and dispose” model to the “reduce, use, recycle and reuse” principles. This principle is regulated by the Federal Project “Circular Economy” in the Russian Federation.

One possible way to transition to a circular economy is through the use of “Green Technologies”. This is an umbrella term for “new” technologies that either do not harm the environment or reduce environmental damage compared to traditional methods and processes. The following “Green Technologies” are applicable to the road construction industry:

1. Reducing noise and vibration levels from transportation [2];
2. Reducing the thickness of pavement structural layers [3];
3. Using eco-friendly resources (bio-bitumen, biofuels, electricity) [4];
4. Using secondary materials (recycling asphalt pavement, fly ash, plastic, waste full, rubber waste) [5].

The use of rubber waste incorporates all aspects of “Green Technologies” applicable to the road industry. This approach not only involves the use of secondary materials but also reduces noise levels and potentially decreases pavement layer thickness. This solution significantly reduces raw material costs during road construction while simultaneously improving quality and contributing to environmental preservation. Thus, many researchers actively consider crumb rubber as an environmentally friendly and economically beneficial modifier for improving bitumen and asphalt concrete properties based on it [6,7]. In recent years, foreign researchers have conducted a large number of studies on rubber-bitumen binders 6–23. It has been established that when optimizing and developing rubber-bitumen binders, the following aspects should be considered: particle size [8,9], amount of additive [10], degree of devulcanization of crumb rubber [11], and physical and chemical properties of base bitumen [12,13]. The performance is also affected by the method of preparation and processing conditions of rubber-bitumen binder [10,11,12,13,14]. Study [14] shows that the content of crumb rubber in bitumen can range from 5 to 20% and can increase resistance to damage caused by water exposure. However, as the crumb rubber content increases further, the overall performance begins to deteriorate. As the particle size of the crumb rubber increases, the complex characteristics of the crumb-rubber-modified binder [15]. However, authors [16] concluded in their study that introducing crumb rubber with a particle size of 2–2.5 mm is the most preferred option. Study [17] showed that devulcanization of crumb rubber can occur due to high-speed shear. Devulcanization allows the crumb rubber to swell and disperse in the maltene part of the bitumen under optimal processing conditions. However, it should be noted that until now, researchers do not have a unified opinion about the optimal processing conditions, and they are determined experimentally in various scientific studies. For example, in study [18], the optimal temperature for preparing crumb-rubber-modified binder was shown to be 180 °C, while authors [6] established the optimal preparation temperature to be 220 °C, and researchers [19] found that temperatures above 240 °C were more preferable for devulcanization of crumb rubber, promoting its more uniform distribution throughout the volume. This also applies to other processing parameters, such as shear stress rate, preparation time, bitumen base, etc. Therefore, establishing or refining the mechanism of interaction between crumb rubber and matrix bitumen remains an urgent direction in this research area. Previously, the authors of the study in the first part of the investigation [20] reviewed and analyzed existing concepts regarding the interaction of crumb rubber particles with bitumen. Therefore, this study continues to explore the structural formation of crumb-rubber-modified binder. Summarizing the results of modern scientific research studying the mechanisms of interaction of components of crumb-rubber-modified binder [21,22,23,24,25] allowed for the formulation of the main phases of the interaction mechanism:

(1) Phase One: Swelling—the particles of the bidding crumb rubber (CR) begin to increase in size by absorbing the light-weight fractions of bitumen. The gelliform layer adjoining the boundary between the oil bitumen fractions and the crumb rubber particles is formed.
(2) Phase Two: Post-swelling and beginning of destruction—the swelling of CR particles continues, accompanied by their chemical destruction due to the breakdown of the vulcanized bonded polymer mesh, resulting in the swollen rubber particles disintegrating into smaller fragments.
(3) Phase Three: Destruction and complete breakdown of—in this phase, the main bonded polymer mesh of the already devulcanized CR particles breaks down until the crumb rubber particles are fully dissolved in the bitumen matrix, resulting in a homogeneous binder.

It should be noted that in previous studies by the authors, it was found that the use of activated crumb rubber from large-sized tires (ACR LST) combined with a compatible hydrocarbon plasticizer did not achieve a sustainable and stable structure of crumb-rubber-modified binder. The activation of crumb rubber typically involves partial surface devulcanization, which makes the surface of the CR more “developed,” as previously demonstrated by [6,7,8] was sufficient for effective interaction with plasticizers and the bitumen base. However, in our experiments, it was found that surface devulcanization alone was insufficient to ensure adequate solubility. Additionally, it was determined that the “hydrocarbon plasticizer—crumb rubber” systems exhibit a supramolecular plasticization mechanism, which is insufficient for obtaining stable, thermodynamically consistent modified asphalt binder. Believed by researchers that it is necessary to first perform partial (controlled) physical devulcanization (bulk) of the crumb rubber to reduce the energy barrier for the subsequent swelling, post-swelling, secondary destruction, and dissolution processes. Therefore, we suggest improving the current understanding of the mechanism of effective interaction with crumb rubber [21,22,23,24,25], as outlined in the analytical section of the study, by introducing an additional initial step: controlled partial physical devulcanization (in bulk) of the crumb rubber beforehand.

This study is aimed at creating a scientifically validated method for targeted thermomechanical devulcanization, which ensures the effective solubility of crumb rubber within the complex structure of a polydisperse composite material. This method will help form a stable, thermodynamically consistent structure in crumb-rubber-modified binders, enhancing the stability and durability of asphalt concrete. At the same time, it will lower construction costs and promote environmental sustainability by recycling used tires.

## 2. Materials and Methods

### 2.1. Raw Materials

The following hydrocarbon plasticizer was considered:

The purified waste frying oil (WFO) is produced by “Promekoglobal” LLC, Moscow, Russia. The production process involves filtering the waste oil through cyclones to eliminate mechanical impurities and water. The purified WFO contains organic non-lauric mono-, di-, and triglycerides, as well as distilled free fatty acids. According to previously calculated semi-empirical compatibility parameters (presented in the first part of the article), this plasticizer is the most compatible. The physical properties of the investigated plasticizer are shown in Table 1.

The following material derived from waste rubber products was considered:

Crumb rubber (CR CRP 0.5) is a crumb rubber produced by “Chekhov Regenerate Plant” LLC, Moscow, Russia and obtained by crushing and grinding pneumatic tires from passenger vehicles with the step-by-step removal of textile, synthetic, and metal cord using an “Eldan” equipment (Faaborg, Denmark) production line.

The following bitumen was considered:

To prepare the crumb-rubber-modified binder, road bitumen of grade BND 70/100, produced by “LUKOIL-Nizhegorodnefteorgsintez” LLC, Nizhny Novgorod, Russia, was used. The bitumen was tested for compliance with the requirements of Russian State Standard GOST 33133-14. The results of laboratory tests of the physical and mechanical properties of the bitumen are presented in Table 2.

### 2.2. Methods

The object under study: the rubber reclaim:

#### 2.2.1. Preliminary Controlled Partial Physical Destruction of Rubber Reclaim (Technology and the Degree of Devulcanization)

**Figure 2:**
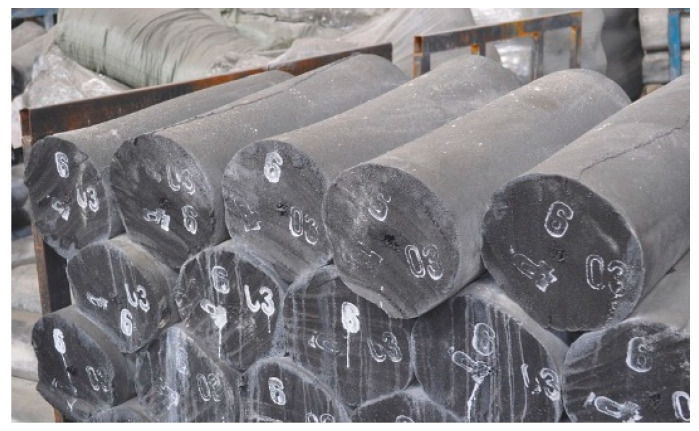
Commercial form of rubber semi-finished product (the size of one roll is 1 m long and 30 cm in diameter).

The destruction process involves breaking disulfide bonds (SS) and carbon-sulfur bonds (C-S), i.e., breaking the cross-linked bonds. If the devulcanization process is uncontrolled, it will result in the uncontrolled degradation of polymer chains, preventing the improvement of binder properties. Researchers [6,18] have also pointed out that chemical degradation has a detrimental impact on the mechanical properties of bitumen binders. Therefore, it was decided to use physical methods for limited destruction, such as high shear force (rollers), high temperature, and a plasticizing medium. In a results investigation of the Russian rubber industry, it was found a semi-finished product obtained from crumb rubber through high shear rolling forces at high temperatures in a fuel oil medium. The commercial appearance is shown in Figure 2.

**Figure 3:**
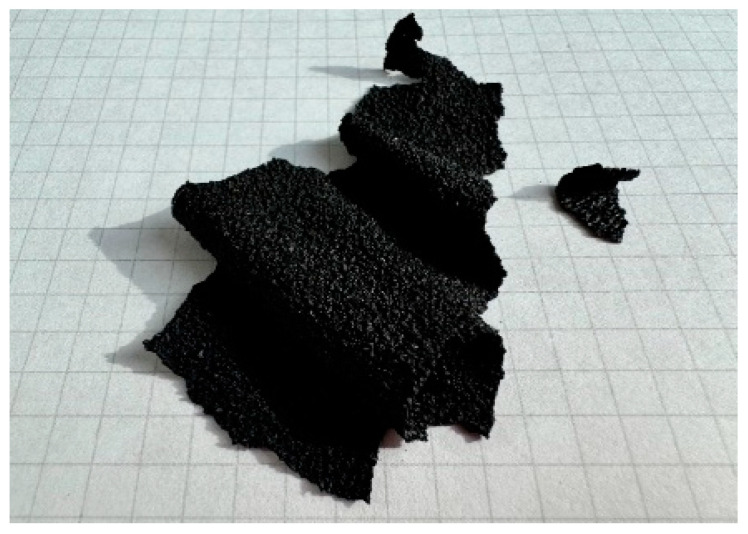
The appearance of the test sample of the rubber reclaim (RR CRP) in the film (the size of one cell is 5 × 5 mm^2^).

The technological processes for preparing the commercial form of the rubber semi-finished product are a trade secret. Traditionally, the rubber semi-finished product is used as a compensator in the construction of tram tracks or is reused in rubber mixtures. As can be seen, in its original form, the rubber semi-finished product cannot be used as a modifying component for bitumen binders due to its tightly compressed roll form. Therefore, it was suggested to take a sample at the intermediate stage of production. During a visit to the production facility, a sample of rubber reclaim (RR CRP) was taken in the form of a film from the production line before the reclaim was formed into rolls, as shown in Figure 3.

##### Technology for Producing Rubber Reclaim (CR CRP)

**Figure 4:**
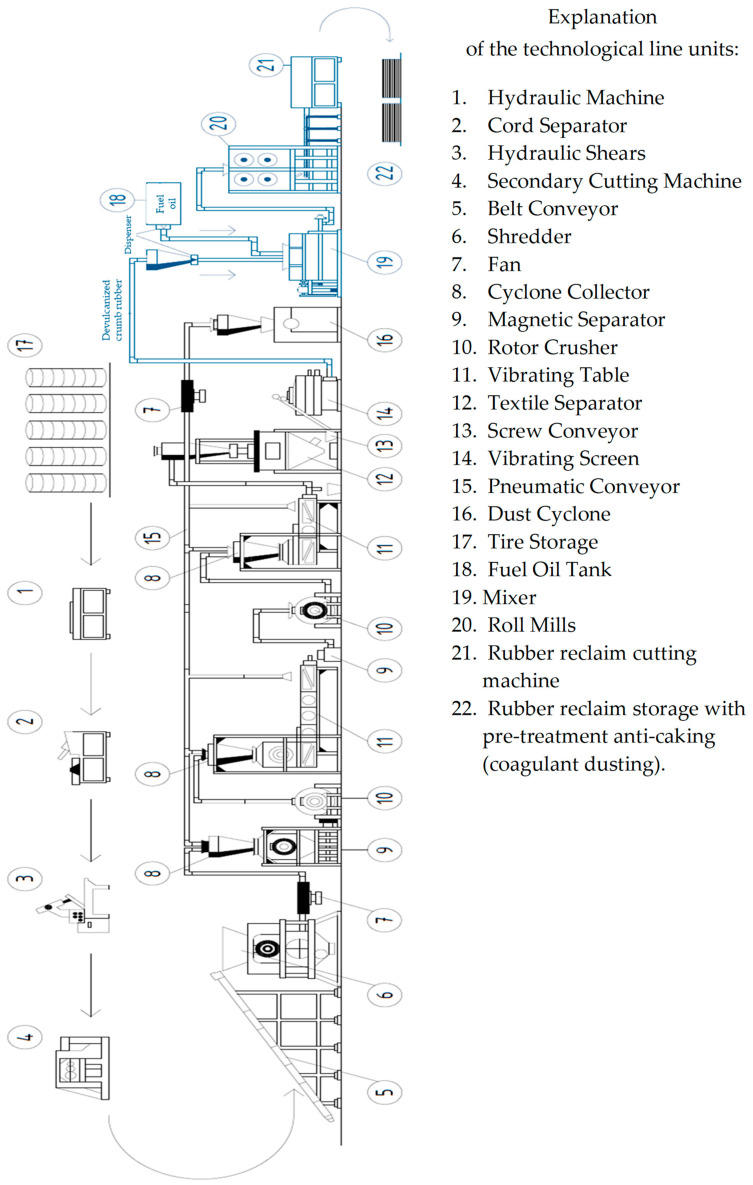
Scheme for processing rubber technical products. Production of rubber reclaim (black—existing technological line; blue—proposed technological line).

After studying and analyzing the existing production of rubber technical products, the authors propose the following technological scheme, shown in Figure 4.

**Figure 5:**
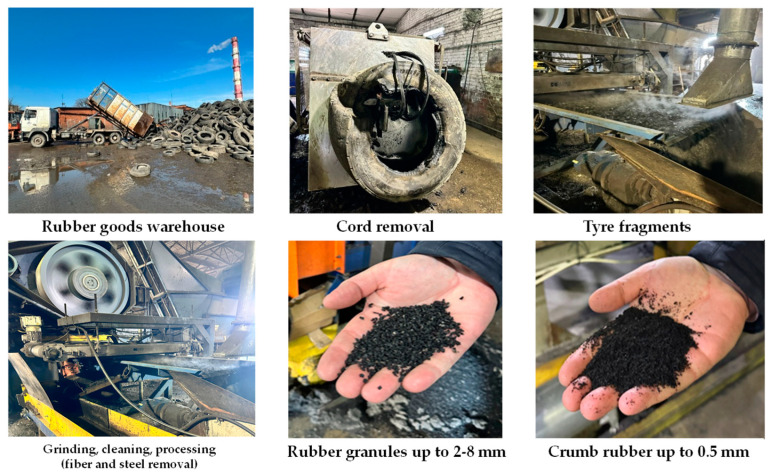
Stages of Processing Rubber Technical Products.

Technology for processing rubber technical products with subsequent production of rubber reclaim. Existing technological line for processing rubber technical products (Figure 5):

1. Collection and transportation of waste tires: used tires are collected from designated areas or collection points and transported to the processing plant.
2. Preparatory work: the waste tires are sorted by size, quality, and other characteristics to ensure that the resulting rubber crumb has a specific homogeneous composition.
3. Cord removal: on a specialized machine (cord separator), the metal and textile cord is removed from the waste tires to reduce wear on cutting equipment during further processing.
4. Shredding of waste tires: after cord removal, the used tires are cut into fragments of 5–15 cm in size using hydraulic shears.
5. Shredding of tire fragments: the tire fragments are conveyed by a belt conveyor into a shredder, where they are ground into granules with sizes ranging from 2 to 5 mm.
6. Cleaning and treatment: the rubber granules are then cleaned and treated to remove dirt, dust, and other non-metallic impurities using a cyclone collector.
7. Removal of metallic components: the cleaned rubber granules pass through a magnetic separator to remove metallic components, including steel rims and spikes.
8. Grinding of rubber granules: the cleaned granules are further processed in a rotor crusher to produce rubber crumb with a size of up to 1 mm. The rubber crumb is then transported via a pneumatic line to a vibrating table, where particles larger than 1 mm are identified and removed. Simultaneously, textile cord, dust, and metallic impurities are also removed. If necessary, the production line can be halted at this stage to discharge the rubber crumb into bulk bags via the pneumatic line or conduct a repeated grinding operation to achieve a crumb size of up to 0.5 mm.

Proposed technological line for rubber reclaim production:

9. Mixing crumb rubber with fuel oil: crumb rubber with a particle size of up to 0.6 mm is transported via pneumatic lines through dispensers into a mixer. Simultaneously, heated fuel oil at a temperature of 70–90 °C is also fed through dispensers into the mixer. The dosage of fuel oil is in the range of 22 ± 2% by mass of the crumb rubber. The rubber mixture is stirred for 10–45 s.
10. Rolling: the rubber mixture is then transported via pneumatic lines to rollers with heated surfaces (surface temperature of 70–80 °C), resulting in the formation of a sheet of rubber reclaim with a thickness of 1–2 mm.
11. Cutting rubber reclaim: the sheet is then conveyed to a rubber reclaim cutting machine, where it is cut into squares measuring 2 × 2 mm with a thickness of 1–2 mm.
12. Dusting and storage: the square samples of rubber reclaim are dusted with an anticoagulant using a ventilation unit equipped with a dispenser and a vibrating screen. The dusted rubber reclaim is then packed into bags weighing 30 or 50 kg.

##### Determination of the Degree of Devulcanization of the Rubber Reclaim

The degree of devulcanization of the rubber reclaim was controlled using the acetone-chloroform extraction method. A sequential extraction using acetone and chloroform was carried out in accordance with the Russian State Standard GOST ISO 1407-2013 using a Soxhlet apparatus, consisting of an extractor, where the reclaim sample wrapped in a paper packet is placed; a heated receiving vessel containing the appropriate solvent where the extracted reclaim extract accumulates; and a condenser where solvent vapors condense. The extraction is carried out continuously for 10 h, after which the receiver is removed from the heating device, the solvent is distilled off from the extractor, and the residue in the vessel is dried in a drying oven, cooled, and weighed. The dry residue weight, expressed as a percentage, is called the acetone or chloroform extract, depending on the solvent used for extraction, as shown in Formula (1):

(1) $$E=\frac{m_{2}}{m_{1}}\times 100,$$

where *E*—the acetone or chloroform extract, %; $m_{1}$—the mass of the rubber reclaim, g; $m_{2}$—the mass of the substance extracted from the rubber reclaim by the corresponding solvent, g.

The acetone extract represents the number of substances extracted from the rubber reclaim by acetone, characterizing the quantitative content of plasticizers in the reclaim. The chloroform extract represents the number of substances extracted from the reclaim by chloroform after removing substances extractable by acetone. Chloroform extracts part of the rubber substance that has gained solubility (rubber degradation products dissolve in chloroform). Thus, the amount of the chloroform extract can be used to assess the degree of degradation (devulcanization) of the original used vulcanized rubber that has undergone regeneration.

The object under study is binders modified with rubber reclaim (BMwRR).

#### 2.2.2. Mathematical Experiment Planning

In order to develop experimental-statistical models that reflect the impact of key compositional and/or technological factors on the properties of the research object, and which are suitable for the subsequent multi-criteria optimization of the crumb-rubber-modified binder production process using the studied rubber reclaim (RR ChRP), a two-factor composite design method of mathematical experiment planning was employed. Throughout the development of the crumb-rubber-modified binder, the bitumen content was maintained at a fixed 100%. The chosen factors were the concentrations of RR CRP (X_1_) and used purified waste frying oil (X_2_). When selecting the range of plasticizer variation, adjustments were made (differing from the plasticizer content in Part I of the article) to reduce its amount, as RR CRP already partially contains a plasticizer. The selected planning parameters and the planning matrix are presented in Table 3 and Table 4.

#### 2.2.3. Methodology for Preparing Bitumen Modified with Rubber Reclaim (BMwRR)

The process begins with weighing the necessary amounts of raw materials (rubber reclaim, hydrocarbon plasticizer, and bitumen). The rubber reclaim is first cut into 5 mm pieces using scissors. The bitumen is preheated to 160 °C in a drying oven. To prepare the bitumen base for modification, the heated bitumen and plasticizer are combined: a high-shear mixer (Silverson L5M-A) equipped with a heat control sensor is immersed in the container, and the system is heated to 175 °C with a stirring speed of 2000 rpm. Once the temperature reaches 175 °C, the stirring speed is increased to 5000 rpm, and the rubber reclaim is added to the bitumen base, followed by mixing for 40 min. In the next step, the crumb-rubber-modified binder undergoes a maturation process by mixing with an IKA EUROSTAR 100 digital stirrer at 300 rpm and 175 °C for 3 h. To complete the polymer network formation, the maturation continues in a drying oven at 175 °C for 18 h, simulating the actual production process. This laboratory treatment of the modified binder simulates real-world transportation to the final consumer. For each method and percentage of rubber reclaim and plasticizer, at least three samples were prepared and tested, with a standard deviation of no more than 2%.

#### 2.2.4. Determination of the Homogeneity, the Solubility, and the Storage Stability of the Obtained Binders Modified with Rubber Reclaim (BMwRR)

The homogeneity of the binders modified with rubber reclaim (BMwRR) was evaluated in accordance with Russian State Standard GOST R 52056-2003. This method involves visually inspecting the binder’s homogeneity using a glass rod. Before testing, the binder is heated to a temperature 10 °C higher than its preparation temperature and stirred for 5–6 min. A glass rod is then dipped into the sample for 3–4 s, removed, and the binder’s flow behavior and the appearance of the film on the rod are visually assessed. The binder should flow smoothly from the rod without any clots, lumps, or granules on its surface. Homogeneity is determined by comparing the results of three trials. If two out of three trials give positive results, the binder is considered homogeneous.

**Figure 6:**
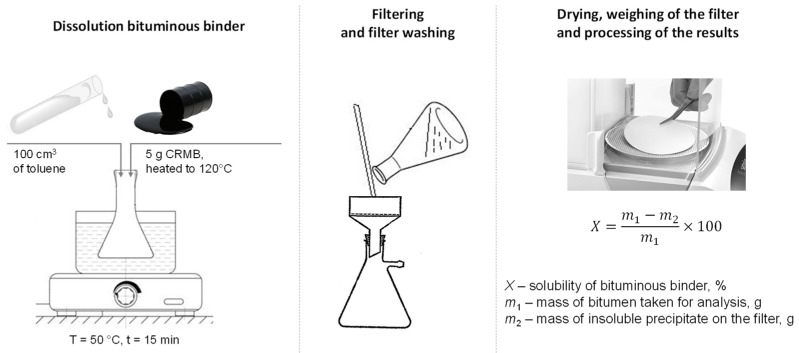
Determination of the solubility of the BMwRR according to Russian State Standard GOST 33135-2014.

The solubility of the obtained binders modified with rubber reclaim (BMwRR) was tested according to Russian State Standard GOST 33135-2014, which measures the degree of solubility in an organic solvent, specifically toluene. A 5 g sample of the crumb-rubber-modified binder is heated in a drying oven at 120 °C. The heated binder is then dissolved in 100 cm^3^ of toluene in a heating flask placed in a water bath at 50 °C and stirred. After 15 min, the solution is filtered through a double-ash-free filter with 2-micron pores. The residue is washed onto the filter using solvent preheated to 50 °C. Once filtration is complete, the filter with sediment is washed with the preheated solvent, transferred to a weighing cup, and dried in an oven for at least 20 min at 20 °C above the solvent’s boiling point. The weighing cup is then covered with a lid, cooled in a desiccator for 30 min, and weighed. A schematic of the testing method is shown in Figure 6.

The solubility of BMwRR was calculated by the formula:

(2) $$X=\frac{m_{1}-m_{2}}{m_{1}}\times 100,$$

where *X*—solubility of the modified binder, %; *m*_1_—the mass of the modified binder taken for analysis, g; *m*_2_—the mass of the insoluble residue on the filter, g.

**Figure 7:**
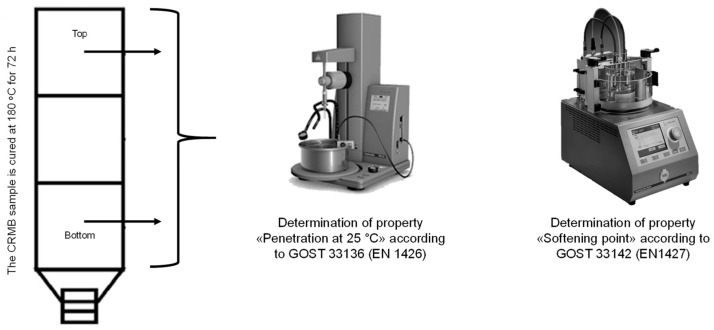
Determination of the storage stability of the BMwRR according to Russian State Standard GOST EN 13399-2013.

The storage stability of binders modified with rubber reclaim (BMwRR) was assessed according to Russian State Standard GOST EN 13399-2013. This method involves placing a homogeneous sample of the modified binder in a vertical tube (100–120 mm in height) and maintaining it at a temperature of 180 °C for three days in drying oven. After cooling, the sample is divided into three equal parts, and the properties of the top and bottom sections are evaluated. In Russia, the properties tested include “Needle penetration depth at 25 °C” in accordance with GOST 33136 (EN 1426) and the “Softening point using the ring-and-ball method” as per GOST 33142 (EN 1427). The difference in these values between the top and bottom sections is then calculated. The binders modified with rubber reclaim are considered stable if the difference in the softening point does not exceed 3 °C and the difference in needle penetration depth at 25 °C is no greater than 20 (0.1 mm). A schematic of this testing method is shown in Figure 7.

#### 2.2.5. Study of the Dependence of the Influence of Formulation Factors on the Structure and Properties of Binders Modified with Rubber Reclaim (BMwRR)

Establishing the dependence of the influence of formulation factors on the structural parameters and properties of binders modified with rubber reclaim is planned to be carried out using the methods specified in regulatory documents governing quality in accordance with Russian State Standard (RSS) GOST R 58400.1-2019 and Russian State Standard GOST R 58400.2-2019, as presented in Table 5.

- The structural stability of the bitumen and modified binders under shear loads and intramolecular interaction was evaluated by the “Dynamic Viscosity” indicator. The determination of dynamic viscosity involves measuring the relative resistance to flow caused by shear stress on the binder by rotating elements of the measurement system of coaxial cylinders with a controlled shear rate. Dynamic viscosity is calculated as the ratio between the applied shear stress and the shear rate (Item 1, Table 5);
- The compliance of the developed binders modified with rubber reclaim with safety requirements was assessed by the “Flash Point” indicator. The determination of the flash point in an open cup using the Cleveland method involves heating a sample of bitumen in an open cup at a set rate until the bitumen vapors flash above its surface from an ignition source (Item 2, Table 5);
- The resistance of the binder to high temperatures and air exposure, simulating technological (short-term) aging processes, was determined using the RTFOT (Rolling Thin Film Oven Test) aging method and subsequent comparison of the physical and chemical properties of bitumen before and after exposure, specifically determining the “Mass Change After Aging” indicator (Item 4, Table 5);
- The resistance of the binder to plastic deformation, contributing to resistance against rutting, was determined by the “Shear Stability G*/sin δ” indicator for the original and RTFOT-aged binder. The method involves assessing the ability of the binders modified with rubber reclaim to resist shear impacts and determining the values of the complex shear modulus G* and the sine of the phase angle δ (Items 3 and 5, Table 5);
- The resistance of the binder under multiple shear deformations (MSCR) is determined by repeated cyclic loading on an RTFOT-aged sample by applying and removing shear stress over a set period and measuring the deformation and elastic recovery of the sample in each cycle (Item 6, Table 5);
- The resistance of the binder to elevated temperatures and pressures, simulating operational (long-term) aging processes during 5 to 10 years of road pavement service, was determined using the PAV (pressure aging vessel) aging method;
- The resistance of the binder to fatigue cracking in asphalt concrete pavement under repeated loads from vehicle traffic was determined by the “Fatigue Resistance G*·sin δ” indicator for the PAV-aged binder (Item 7, Table 5);
- The resistance of the binder to cracking at low service temperatures was determined by the “Cracking Temperature” indicator. The method involves cooling a sample of the bituminous binder in the shape of a ring and recording the jump in deformation (Item 8, Table 5);
- The upper grade value X, corresponding to the maximum allowable operating temperature at which the binder modified with rubber reclaim retains its properties, is assigned based on the results of the above tests;
- The lower grade value Y, corresponding to the minimum allowable operating temperature at which the binder modified with rubber reclaim retains its properties, is assigned based on the results of the above tests.

## 3. Results and Discussion

The object under study: the rubber reclaim.

### 3.1. Preliminary Controlled Partial Physical Destruction of Rubber Reclaim

The preliminary partial destruction of the rubber reclaim means ensuring the devulcanization of rubber before being introduced into bitumen at a certain level. That is, it is necessary to maximally destroy the transverse, most often C-S and S-S bonds, while preserving the rubber molecule from thermal degradation as much as possible. Believed by researchers that it should not be less than 20% and not more than 50%. This will make it possible to obtain a rubber reclaim with elasticity and plasticity. With the acetone-chloroform extraction method, it was established that the degree of devulcanization in the studied sample of rubber reclaim is 22%. To determine the stability of this indicator over time (i.e., the criterion for the controllability of destruction), this indicator was measured after 1, 7, and 14 days; the results are presented in Table 6.

The analysis of the obtained data (Table 6) shows that the variation in the degree of vulcanization of the studied rubber reclaim RR CRP over time is at the level of 3%, indicating the stability of the obtained values at different time intervals, which allows concluding the observed controlled limited destruction over 14 days. Believed by researchers that the stability of the degree of destruction of the rubber reclaim is achieved by the fact that it is selected in the form of a thin film in hot conditions. Then the thin film is subjected to a fast cold-cooling process in the chamber until it reaches room temperature. However, it is worth noting that research in this area continues.

The object under study: of binders modified with rubber reclaim (BMwRR).

### 3.2. Determination of the Homogeneity, the Solubility, and the Delamination of the Obtained Binders Modified with Rubber Reclaim (BMwRR)

According to the chosen experimental design, nine compositions of binders modified with rubber reclaim were prepared (Table 3 and Table 4). The key properties determining the effectiveness of these compositions include the homogeneity of RR CRP distribution, as well as solubility and storage stability, which reflect the binder’s overall durability. The solubility indicator is the most crucial characteristic, as per Russian regulations, binders modified with rubber reclaim cannot be used in road construction without meeting this requirement. The result of rubber reclaims impact on the structural stability of the modified binders is shown in Table 7.

According to the obtained data (Table 7), it can be concluded that several samples of BMwRR (binders modified with rubber reclaim) met all the necessary parameters for homogeneity, solubility, and stability. More than 99% modified binder of samples No. 10, 11, 12, 15, and 18 passed through 2 μm cell size filters. This means that the size of rubber reclaim particles less than 2 μm is reached. Which meets the requirements of the state standard for solubility. Compliance with the solubility index allows us to conclude that part of the rubber has passed into the liquid phase of bitumen. Which, in accordance with [18], will have a positive effect on storage stability. Regarding the storage stability, assessed by the segregation of the binder kept for 3 days at 180 °C, all studied samples showed similar good values. Thus the difference in the softening point was from 0.3 to 0.5 °C, and the penetration depth was from 1.5 to 2.2 mm⁻¹. This indicates minor changes in properties and storage stability of modified binders. At this stage of the research, it is not possible to choose the most stable composition modified with rubber reclaim. Therefore, for further research on establishing the dependence of formulation factors on the structural parameters and properties of the binder, all compositions (No. 10, 11, 12, 15, and 18) were considered.

### 3.3. Dependence of the Influence of Formulation Factors on the Structure and Properties of Binders Modified with Rubber Reclaim (BMwRR)

The results of the influence of different percentages of rubber reclaim and purifying waste frying oil on the structural stability of the binder under shear loads, plastic deformation, fatigue cracking, transverse cracking during cooling, and the operational temperature range of the binder (PG grade) are presented in Table 8.

When developing an effective composition of a binder modified with rubber reclaim, the primary goal was to achieve the lowest possible operating temperature during the winter season. This is because bituminous binders containing rubber waste often form aggregates consisting of non-wetted polymer particles of rubber crumb, leading to elastic aftereffects that cause intense cracking, especially during the low-temperature periods of road operation. The developed binder is planned to be used in the construction of the federal highway M-12 on a section passing through the Yekaterinburg region, Russia. For this region, the upper grade value X, according to the PG classification, corresponding to the maximum allowable operating temperature at which all properties of the binder are retained, is 64 °C. According to the requirements of Russian State Standard GOST R 58400.2, the lower grade value Y, corresponding to the minimum allowable operating temperature in this case, is −40 °C. Therefore, in our study, it is necessary to develop the binder modified with rubber reclaim with a performance grade of PG 64-40. Without the use of additional adhesive additives and cross-linking agents.

**Figure 8:**
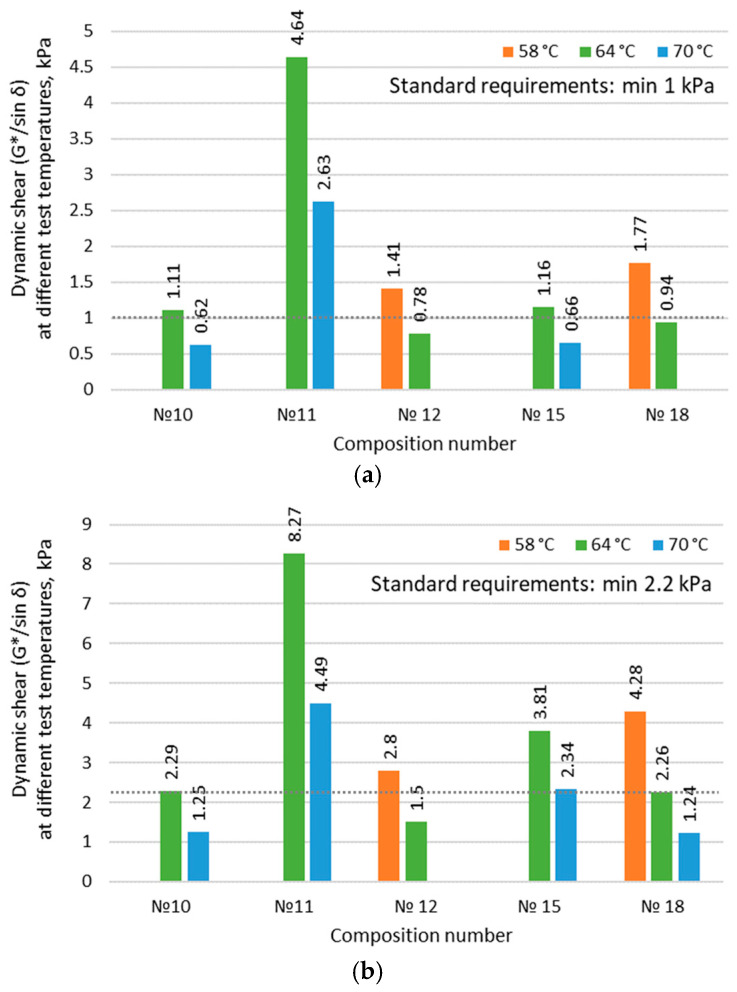
Influence of the component composition of binder modified with rubber reclaim on shear stability at various test temperatures: (**a**) original binder; (**b**) RTFOT binder.

The analysis of the obtained data (Table 8) allows us to conclude that the dynamic viscosity at 130 °C for all binder samples modified with rubber reclaim is less than 2 Pa·s. Evidence that the use of rubber reclaim will not cause technological difficulties in the production of industrially modified binders that would increase costs. The study of fundamental rheological indicators, such as the shear stability of the original and RTFOT binders, allows us to assess the ability of the binder modified with rubber reclaim to resist shear stresses and predict the intensity of rutting in the road pavement. The results are presented in Figure 8.

As a reminder, according to the research objective, it is important to develop a binder that will effectively resist dynamic shear, leading to plastic rutting formation at 64 °C. As seen (Figure 8, green color), modified binder compositions No. 10, 11, and 15 meet these requirements.

The maximum positive operating temperature of the modified binders (PG X) is determined by taking the maximum temperature at which the shear stability of the original binder and the RTFOT binder is above 1 kPa and 2.2 kPa, respectively, according to the requirements of Russian Satet Standard GOST 58400.1 (Table 9).

The best values for shear stability (Table 8, Figure 7) correspond to binder samples No. 11 and No. 15. The binder compositions differ only in terms of plasticizer content. The lower plasticizer content in composition No. 11 (by 20% less) naturally led to higher shear stability values.

**Figure 9:**
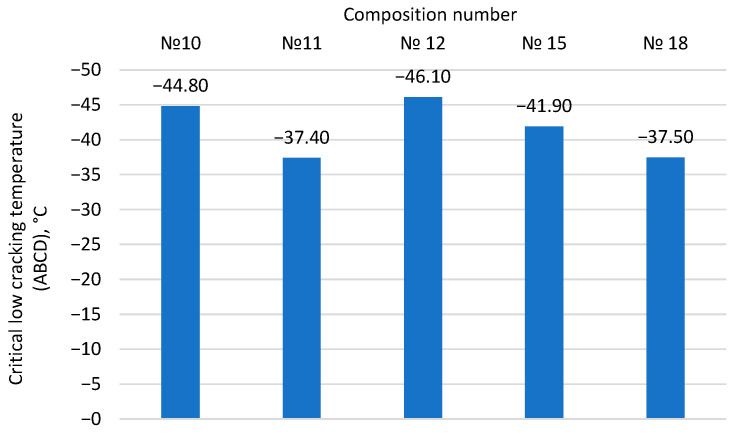
Influence of the component composition of binder modified with rubber reclaim on the cracking temperature according to the ABCD method.

The lower grade of the binder (PGY) predicts the low-temperature crack resistance of asphalt concrete. It is determined by the ABCD method based on the cracking temperature, which corresponds to the jump in deformation in the binder aged by the PAV method during cooling. The results are presented in Figure 9.

As shown, binder samples No. 10, 12, and 15 meet the set goal of the minimum allowable operating temperature of −40 °C. In contrast, composition No. 11, which previously showed the best results in shear stability, does not meet the final target requirements. In this case, the minimum allowable temperature was −34 °C. Composition No. 18 also showed unsatisfactory values.

**Figure 10:**
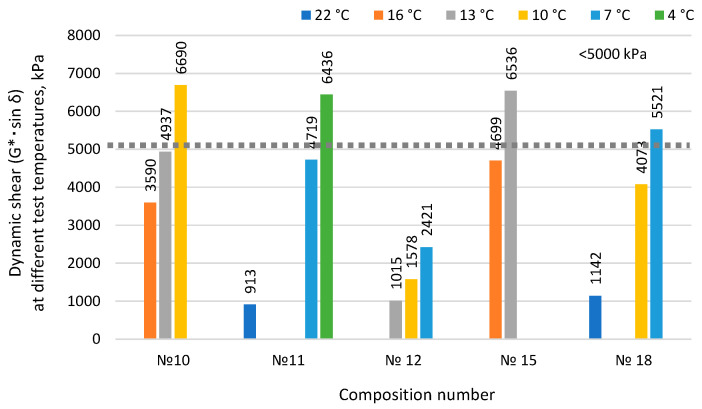
Influence of the component composition of binder modified with rubber reclaim on fatigue resistance.

When developing binder modified with rubber reclaim, it is important to also study indicators that characterize durability. One of these indicators is “Fatigue Resistance (G*·sin δ)” for binders aged by the PAV method. This indicator characterizes resistance to fatigue cracking in asphalt concrete. The results of determining fatigue resistance and the temperatures of binders associated with their fatigue failure are presented in Figure 10.

The measured values of the complex shear modulus G* and the phase angle δ characterize viscoelastic properties. According to Russian State Standard GOST R 58400.1-2019, the value of their multiplication G*sin δ should not exceed 5000 kPa for the modified binder, exhibits viscoelastic properties, and is recovered after load-off. That is, it should not be too tough. This is very often seen in bitumen that contain crumb rubber [26]. But it has a negative impact on fatigue resistance [27]. The binder modified with rubber reclaim No. 15 has the highest fatigue resistance value (Figure 10) at 4699 kPa, indicating that after the effects of long-term PAV aging, the modified binder can effectively exhibit viscoelastic properties and recover after load-off. This characterizes the modified binder as a system with a rubber reclaim network resistant to long-term aging processes.

**Figure 11:**
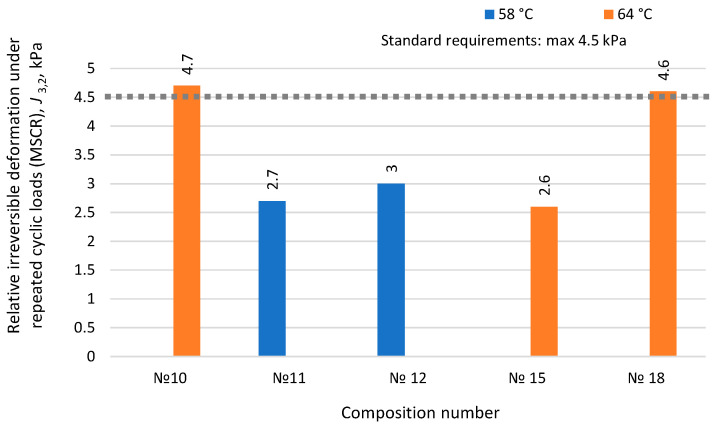
Influence of the component composition of binder modified with rubber reclaim on relative irreversible deformation under repeated cyclic loads.

The MSCR test allows the evaluation of the elastic properties of the studied binders by their resistance to repeated cyclic impacts through the application and removal of shear loads over a certain period. It is assessed by determining the *J*_3,2_ average value of relative irreversible deformation from ten test cycles (creep phase—recovery phase) at high temperature. The lower the *J*_3,2_ value, the more elastic the binder is at high temperatures, i.e., it can restore its structure after load removal. The results of determining the resistance of the developed binders to repeated shear deformations are presented in Figure 11.

The obtained average values of relative irreversible deformation under repeated cyclic loads characterize the binders’ resistance to rutting. According to the data binder modified with rubber reclaim, No. 11 and 15 (containing 10% rubber reclaim) showed the lowest *J*_3,2_ values, indicating the high operational resistance of the developed binders to shear loads at high temperatures (64 °C). The MSCR test results also determine the grade type of the developed modified binders corresponding to the permissible level of traffic load. The results for grade type determination based on the allowable load are presented in Table 10.

Test results show that compositions No. 11, No. 12, and No. 15 correspond to type S for the permissible traffic load level but at different operating temperatures. The load level of type S is appropriate for normal conditions of traffic. It means that the modified binder can be used on roads with traffic intensity less than 1.8 million per day and average speed of traffic greater than 70 km/h. Compositions No. 10 and No. 18 do not meet the requirements for resistance to repeated shear deformations (Table 8) and cannot effectively resist dynamic shear from vehicles.

Summarizing the results of the development of the binder modified with rubber reclaim (Table 8, Table 9 and Table 10, Figure 7, Figure 8, Figure 9, Figure 10 and Figure 11), it can be concluded that the most promising and optimal composition is No. 15, containing 10% rubber reclaim and 7.5% plasticizer, corresponding to grade PG 64(S)-40. It is also worth noting that composition No. 11, containing 10% rubber reclaim but a lower plasticizer content of 6%, showed high results in shear and fatigue resistance and high-temperature operational limits. Based on this, it can be concluded that the optimal content of rubber reclaim in the studied binders is 10%, and by varying the plasticizer content, the desired characteristics of the modified binders can be achieved.

The plan for further research will include the following points: studying the features of intermolecular interactions in binder samples with rubber reclaim, specifically where the necessary solubility indicators were achieved, in comparison with binder samples containing crumb rubber, where these were not achieved (compositions of crumb-rubber-modified binders from the first part of the article [20]) using infrared spectroscopy. Stability parameters (homogeneity, solubility, particle size) of the modified binders will also be studied at different time intervals. The thermodynamic compatibility parameter for the “rubber reclaim-frying oil” dispersed system will be determined. Additionally, further optimization of the composition of the binder modified with rubber reclaim, frying oil, and other hydrocarbon plasticizers will be carried out. The durability parameters of the modified binders and the asphalt concrete based on them will also be determined. The asphalt concrete tensile strength module will also be determined experimentally. The road design will be calculated on the basis of these data. To establish the possibility of reducing the thickness of the layers.

## 4. Conclusions

A scientifically based technological method for targeted thermomechanical devulcanization has been developed and proposed, which ensures the solubility of crumb rubber within the complex structure of a polydisperse composite material. The authors expanded upon existing theories of effective interaction with crumb rubber by introducing an initial stage: controlled partial physical destruction of the rubber crumb to produce rubber reclaim. This step ensures the solubility of crumb rubber, preventing the formation of large aggregates of rubber particles. That contributes to premature crushing of the asphalt concrete pavement. The preliminary controlled partial physical destruction of crumb rubber includes the following stages: high temperature and a plasticizing medium (fuel oil) to produce rubber reclaim (RR CRP). Controlled partial destruction was evaluated using the acetone-chloroform extraction method. It was established that the degree of devulcanization in the studied rubber reclaim sample is 22 ± 0.24%. The change in this indicator within 14 days is not more than 3%. What indicated the achievement of a controlled degree of devulcanization during this period? However, it should be noted that additional studies with a long-time interval are required.

It was found that in samples of binder containing 8 to 10% rubber reclaim and 6 to 9% waste oil, the solubility of the particles of rubber reclaim was more than 99%. The particle size of rubber reclaim. Storage stability was assessed by the separation of the binder maintained for 3 days at 180 °C, and all studied samples show similar values. The softening point differences range from 0.3 to 0.5 °C, and the needle penetration differences range from 1.5 to 2.2 mm^−1^.

Rheological parameters of the structure characterizing intermolecular interactions, such as shear stability for the original and RTFOT-aged binders, are in the range of 1.16 to 4.64 kPa (>1 kPa) at 64 °C and 2.29 to 8.27 kPa (>2.2 kPa) at 64 °C, respectively. The elastic structural component of the binder modified with rubber reclaim (creep-recovery phase), determined by the relative irreversible deformation parameter *J*_3,2_, is no more than 2.6 kPa (<4.5 kPa) at 64 °C. The parameter of fatigue resistance, which characterizes the durability and reliability of road surfaces under intensive use and is evaluated under cyclic bending loads simulating real operating conditions, does not exceed 4699 kPa (<5000 kPa) at 16 °C.

The results obtained from the analysis can be used to conclude that the optimal composition is containing 10% rubber reclaim and conforms to grade PG64(S)-40. It was considered that by varying the purified waste frying oil content, the necessary characteristics of modified binders can be achieved.

## Figures and Tables

**Table 1:** Physical Properties of the Purified Waste Frying Oil.

Indicator Name	Actual Indicators for PWFO
Kinematic viscosity at 50 °С, mm^2^/с	32.99
Kinematic viscosity at 100 °С, mm^2^/с	8.46
Flash point, °С	282
Environmental status (carbon footprint)	net zero
Density at 20 °C, kg/m^3^	908–928

**Table 2:** Physical and mechanical properties of bitumen BND 70/100.

Indicator Name	The Requirements of Russian State Standard GOST 33133-14	Actual Indicators
Needle penetration depth, 0.1 mm, at a temperature of 25 °C	71–100	82
Needle penetration depth, 0.1 mm, at a temperature of 0 °C	more 21	26
Softening temperature of the ring and ball, °C	more 47	48.4
Ductility, cm, at 0 °С	more 3.7	4.1
Fragility, °С	less −18	−20
The change in the mass of the sample after aging, %	less 0.6	0.28
Change in the softening temperature of the sample after aging, %	less 7	6

**Table 3:** Selected planning parameters for developing the composition of crumb-rubber-modified binder with RR CRP and purified waste frying oil.

The Name of the Factor	The Main Level, Х_0_	Interval ΔХ
Х_1_	9	1.0
Х_2_	7.5	1.5

**Table 4:** Planning matrix for developing the composition of modified binder with RR CRP and purified waste frying oil.

Composition Number	In Encoded Values		In Natural Values	
	Х_1_	Х_2_	Х_1_	Х_2_
10	−1	−1	8	6
11	1	−1	10	6
12	−1	1	8	9
13	1	1	10	9
14	−1.41	0	7.59	7.5
15	1.41	0	10	7.5
16	0	−1.41	9	5.38
17	0	1.41	9	9.62
18	0	0	9	7.5

**Table 5:** Methods and devices for measuring the physical, chemical, and performance indicators of bituminous and binder modified with rubber reclaim.

No.	Indicator Name	Test Method	Measuring Instrument
Original Binder			
1	Viscosity at 135 °C	RSS GOST 33137	Rotary rheometer Rheolab QC 18318 Anton Paar
2	Flash point temperature	RSS GOST 33141	Cleveland flash point and ignition analyser Anton Paar CLA5
3	Dynamic shear (G*/sin δ) at test temperature (t, °C)	RSS GOST R 58400.10	Dynamic shear rheometer Malvern Kinexus DSR+
Binder under RTFOT aging process			
4	Mass change	RSS GOST 33140	Cooper Asphalt Rolling Thin Film Oven, scale VIBRA AJH-620CE
5	Dynamic shear (G*/sin δ) at test temperature (t, °C)	RSS GOST 33140 RSS GOST R 58400.10	Dynamic shear rheometer Malvern Kinexus DSR+
6	Stability under multiple shear stress (MSCR)	RSS GOST R 58400.6	Dynamic shear rheometer Malvern Kinexus DSR+
Binder under PAV aging process			
7	Dynamic shear (G*·sin δ) at test temperature (t, °C)	RSS GOST R 58400.5, RSS GOST R 58400.10	CRT-PAV bitumen ageing machine, Dynamic shear rheometer Malvern Kinexus DSR+
8	Critical low cracking temperature (ABCD)	RSS GOST R 58400.11	ABCD EZ Asphalt Technology

**Table 6:** Degree of devulcanization of rubber reclaim at different time intervals.

Object of Study	Degree of Devulcanization, %, at Different Time Intervals, Days				
	0	1	7	14	Standard Deviation
RR CRP	22	22.3	22.5	22.5	0.24

**Table 7:** Results of determining the properties characterizing the homogeneity and stability of the binders modified with rubber reclaim.

Composition Number	Homogeneity, Russian State Standard GOST R 52056	Solubility, % Russian State Standard GOST 33135-2014 (>99%)	Storage Stability, Russian State Standard GOST EN 13399-2013, the Difference in:	
			Softening Point, °С (˂3 °С)	Penetration at 25 °C, °С (˂20, 0.1 mm)
10	homogeneously	more than 99	0.3	1.6
11	homogeneously	more than 99	0.5	2.2
12	homogeneously	more than 99	0.4	2.1
13	heterogeneous	more than 99	-	-
14	heterogeneous	more than 99	-	-
15	homogeneously	more than 99	0.4	2.0
16	heterogeneous	more than 99	-	-
17	heterogeneous	more than 99	-	-
18	homogeneously	more than 99	0.3	1.5

**Table 8:** Influence of formulation factors on the physical, chemical, and performance characteristics of binders modified with rubber reclaim.

Indicator Name	Actual Indicators for Compositions					GOST R 58400.1, GOST R 58400.2, (PG 64-40)	Test Method
Composition number	10	11	12	15	18		
RR ChRP, %	8	10	8	10	9		
PWFO, %	6	6	9	7.5	7.5		
Original Binder							
Viscosity at 135 °C, Pa·s	0.76	1.86	0.63	0.78	0.52	max 3 Pa·s	RSS GOST 33137
Dynamic shear (G*/sin δ) at test temperature (t, °C), kPa	64: 1.11 70: 0.62	64: 4.64 70: 2.63	58: 1.41 64: 0.78	64: 1.16 70: 0.66	58: 1.77 64: 0.94	min 1 kPa at 64 °С	RSS GOST R 58400.10
Binder under RTFOT aging process							
Mass change, %	0.63	0.77	0.86	0.83	0.69	max ±1%	RSS GOST 33140
Dynamic shear (G*/sin δ) at test temperature (t, °C), kPa	64: 2.29 70: 1.25	64: 8.27 70: 4.59	58: 2.80 64: 1.50	64: 3.81 70: 2.34 76: 1.44	58: 4.28 64: 2.26 70: 1.24	min 2.2 kPa at 64 °С	RSS GOST 33140, RSS GOST R 58400.10
Stability under multiple shear stress for type S grade (MSCR), *J*_3,2_ mean value of relative irreversible deformation	4.7 at 64 °С	2.7 at 58 °С	3.0 at 58 °С	2.6 at 64 °С	4.6 at 64 °С	*J*_3,2_ max 4.5 kPa^−1^ at 64 °С	RSS GOST R 58400.6
Binder under PAV aging process							
Dynamic shear (G*·sin *δ*) at test temperature (t, °C), kPa	16: 3590; 13: 4937;	22: 913; 7: 4719;	13: 1015; 10: 1578;	16: 4699; 13: 6536	22: 1142; 10: 4073;	max 5000 kPa at 16 °С (for type S grade)	RSS GOST R 58400.10
Critical low cracking temperature (ABCD), °C	−44.8	−37.4	−46.1	−41.9	−37.5	max −40 °С	RSS GOST R 58400.11

**Table 9:** PG X grade value of the developed binders modified with rubber reclaim.

Indicator Name	Actual Indicators for Compositions				
Composition number	10	11	12	15	18
RR CRP, %	8	10	8	10	9
PWFO, %	6	6	9	7.5	7.5
PG X	64	70	58	64	58

**Table 10:** Influence of components of binder modified with rubber reclaim on grade type by permissible traffic load level.

Indicator Name	Actual Indicators for Compositions:				
Composition number	10	11	12	15	18
RR ChRP, %	8	10	8	10	9
PWFO, %	6	6	9	7.5	7.5
Grade type by permissible traffic load level	not correspond	not correspond/S	S	S	not correspond
Performance Grade, PG Х(Z)-Y	64(-)-40	70(-)-34 58(S)-34	58(S)-40	64(S)-40	64(-)-34

## Data Availability

The original contributions presented in the study are included in the article, further inquiries can be directed to the corresponding authors.
